# Study on the Mechanical Property Degradation Laws of 6061-T6 Aluminum Alloy Under the Synergistic Effect of Corrosion and Cyclic Loading

**DOI:** 10.3390/ma19071416

**Published:** 2026-04-02

**Authors:** Qisheng Long, Xiangjie Nie, Chuanfu Yan, Zhongquan Chen, Zuodong Li, Siru Chen, Zhen Huang

**Affiliations:** 1 School of Civil Engineering, Tianjin University, Tianjin 300350, China; huayejishuzhongxin@163.com; 2 Guangxi Huaye Construction Co., Ltd., Fangchenggang 538021, China; ycf20250225@163.com (C.Y.); chenzhongquan2024@163.com (Z.C.); lizdong2024@163.com (Z.L.); 3 School of Civil Engineering and Architecture, Guangxi University, Nanning 530004, China; chensiru20240210@163.com (S.C.); hzcslg@163.com (Z.H.)

**Keywords:** 6061-T6 aluminum alloy, salt spray corrosion, cyclic loading, mechanical properties, hysteretic performance, energy dissipation coefficient

## Abstract

To investigate the mechanical property degradation laws of 6061-T6 aluminum alloy under the synergistic effect of coastal corrosive environments and cyclic loading, the effects of various corrosion durations (0 h, 600 h, 900 h, and 1200 h) on the static performance, hysteretic characteristics, and energy dissipation capacity of the material were studied through indoor accelerated salt spray corrosion tests, monotonic tensile tests, and multi-regime cyclic loading tests. The results indicate that after 1200 h of corrosion, the yield strength and ultimate strength decreased by an average of 2.28% and 5.16%, respectively, with the peak stress point shifting significantly forward. Corrosion significantly inhibits the cyclic hardening effect and accelerates the loss of ductility, with the ductility loss of 1200 h specimens reaching up to 44.0%. Strain is the key factor in activating the energy dissipation potential of the material; when the loading amplitude exceeds 4%, the energy dissipation coefficient stabilizes between 3.0 and 3.3. However, the combination of corrosion and random loading exacerbates the decay of energy dissipation capacity. This study aims to provide a theoretical foundation for the performance assessment and safety assurance of aluminum alloy structures in coastal engineering.

## 1. Introduction

High-strength aluminum alloys, as lightweight and high-strength structural materials, have become key components in fields such as aerospace, shipbuilding, bridge engineering, and architectural structures, owing to their excellent specific strength, good workability, and outstanding corrosion resistance [1,2]. However, infrastructure and equipment serving in coastal regions face a dual challenge: on one hand, they must withstand continuous corrosive attack triggered by the high-salt and high-humidity environments of the marine atmosphere [3]; on the other hand, they must cope with the alternating internal structural forces caused by cyclic loads such as wind, waves, and seismic actions. The coupling effect of corrosive environments and cyclic loading significantly accelerates the damage evolution process in aluminum alloy materials, leading to a marked degradation of hysteretic performance and a substantial decrease in fracture resistance [4]. This directly threatens the long-term durability and service safety of the structures. Global economic losses caused by marine environmental corrosion are immense; among accidents involving aluminum alloy structures, the vast majority are directly related to the synergistic effect of corrosion and dynamic loading. According to 2024 public data from the Chinese Society for Corrosion and Protection, the annual growth rate of corrosion failure cases in coastal aluminum alloy structures continues to rise, with nearly 60% of these cases involving the superimposed effect of fatigue damage induced by cyclic loading [5,6,7,8,9,10]. The aforementioned cases fully demonstrate that the failure mechanism of aluminum alloy structures under coupled coastal corrosion and cyclic loading has become a critical issue restricting the safe and long-term operation of relevant infrastructure.

In the field of corrosion research, scholars both domestically and internationally have progressed from studying evolution in single environments to investigating the complex mechanisms of multi-factor interactions. Tian et al. [11] explored the atmospheric corrosion evolution patterns and mechanical property degradation characteristics of 2524-T3 aluminum alloy in marine environments. Building upon this, researchers further introduced the coupling effects of physical and chemical fields. For instance, Zhou et al. [12] systematically analyzed the damage behavior of 7A04 aluminum alloy under the interaction of static tensile stress and thin liquid films in real marine atmospheric environments, while Tian et al. [13] elucidated the influence of different pH values on the tribocorrosion evolution of 7075-T651 aluminum alloy. To mitigate corrosion damage in harsh environments, related studies have also explored pathways to enhance the corrosion resistance of materials through processing techniques. These primarily include the use of Ultrasonic Impact Treatment (UIT) to optimize the microstructure of welded joints [14] and thermomechanical processing strategies combining cyclic strengthening with subsequent aging treatments [15]. These efforts aim to achieve a synergistic balance between the mechanical properties and environmental stability of high-strength aluminum alloys.

Regarding the mechanical response under cyclic loading, research has focused on the damage accumulation effects induced by different loading protocols. Wu et al. [16] tested AAL joint specimens using electronic servo-actuators, detailing the failure modes and ultimate load-carrying capacities under cyclic loads. Lu et al. [17,18] conducted comparative tests on A380 and ADC12 cast aluminum alloys, revealing the degradation patterns of peak stress, flow stress, and elastic modulus with cumulative plastic strain, thereby demonstrating a strong correlation between cyclic behavior and strain amplitude. In the realm of structural performance assessment and optimization, scholars have proposed a series of theoretical and numerical models. Kwon et al. [19] utilized ultrasonic waves and relative strain measurement techniques to achieve effective estimations of the variable-amplitude fatigue life for notched aluminum plates. Meanwhile, Pankaj et al. [20] employed a 3D elastoplastic finite element model to quantitatively investigate the influence of parameters such as insertion thickness and edge-margin ratio in cold-expansion strengthening on the residual stress field and crack initiation cycles of aluminum holes. These studies provide critical data support for the durability design of aluminum alloy structures under complex loading conditions.

However, existing research has largely focused on the influence of single factors—either corrosion or cyclic loading—on the performance of aluminum alloys. Under the synergistic effect of high-salinity corrosive environments and complex cyclic loading, the intrinsic correlation mechanisms of the macroscopic mechanical behavior and the evolutionary pathways of performance degradation in aluminum alloys remain to be clarified. This study focuses on 6061-T6 aluminum alloy, a material commonly used in coastal engineering. Through indoor accelerated salt spray corrosion tests, monotonic tensile tests, and multi-regime cyclic loading tests, the influence of corrosion duration on the static and hysteretic performance of the material is investigated. The objective is to provide a reliable data reference for the long-term service performance assessment and the development of protection strategies for aluminum alloy structures.

## 2. Experimental Program

### 2.1. Specimen Preparation and Experimental Conditions

**Figure 1:**
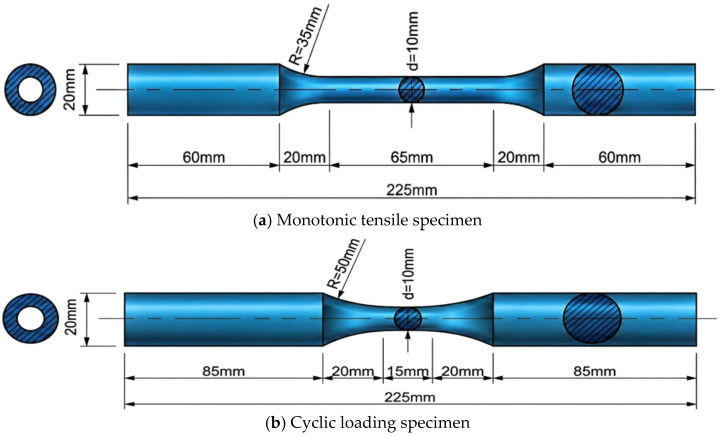
Dimensions of specimens.

This study investigates 6061-T6 aluminum alloy, the chemical composition of which is listed in Table 1. A total of 40 standard specimens were prepared for tests across four corrosion durations: 0 h, 600 h, 900 h, and 1200 h. Each corrosion group included two replicate monotonic tensile specimens and six specimens subjected to different cyclic loading regimes (one for each regime), as detailed in Table 2. The specimen dimensions are shown in Figure 1. To prevent premature buckling under compression during cyclic loading, the specimen geometry was designed in accordance with the Chinese standard Metallic materials—Fatigue testing—Axial strain-controlled method [21].

**Table 1:** Chemical composition of 6061-T6 aluminum alloy.

Element	Si	Fe	Cu	Mn	Mg	Cr	Zn	Ti
GB50429	0.4–0.8	≤0.7	0.15–0.40	≤0.15	0.8–1.2	0.04–0.5	≤0.25	≤0.15
Measured value	0.645	0.488	0.232	0.074	1.044	0.104	0.130	0.025

**Table 2:** Design of experimental conditions.

Corrosion Duration (h)	0	300	600	900	1200
Monotonic tension	ML-0-1	ML-300-1	ML-600-1	ML-900-1	ML-1200-1
	ML-0-2	ML-300-2	ML-600-2	ML-900-2	ML-1200-2
Constant amplitude loading	CL-DC-A-0	/	CL-DC-A-600	CL-DC-A-900	CL-DC-A-1200
	CL-DC-B-0	/	CL-DC-B-600	CL-DC-B-900	CL-DC-B-1200
	CL-SC-A-0	/	CL-SC-A-600	CL-SC-A-900	CL-SC-A-1200
Variable amplitude loading	CL-DC-D-0	/	CL-DC-D-600	CL-DC-D-900	CL-DC-D-1200
	CL-DC-E-0	/	CL-DC-E-600	CL-DC-E-900	CL-DC-E-1200
	CL-SC-D-0	/	CL-SC-D-600	CL-SC-D-900	CL-SC-D-1200
Random loading	CL-DC-G-0	/	CL-DC-G-600	CL-DC-G-900	CL-DC-G-1200
	CL-DC-H-0	/	CL-DC-H-600	CL-DC-H-900	CL-DC-H-1200

Note: In Table 2, ML represents monotonic tensile loading, while CL denotes cyclic loading. Among the cyclic loading specimens, DC indicates displacement-controlled loading and SC signifies strain-controlled loading. The subsequent letters A, B, D, E, G, and H represent six different cyclic loading regime designs, with specific details regarding the cyclic loading amplitudes provided in Table 3. The numerical values in the specimen IDs are used to identify the corrosion duration. For instance, CL-DC-A-600 represents a specimen subjected to 600 h of corrosion and tested under constant-amplitude-displacement-controlled cyclic loading.

**Table 3:** Design of cyclic loading regimes.

Loading Regimes	Description of Cyclic Loading Amplitudes
CL-DC-A	Constant-amplitude displacement: ±0.15 mm, ±0.3 mm, ±0.6 mm, ±0.75 mm; each amplitude is repeated 4 times.
CL-DC-B	Constant-amplitude displacement: ±0.15 mm, ±0.375 mm, ±0.3 mm, ±0.525 mm, ±0.445 mm, ±0.675; each amplitude repeated 4 times.
CL-DC-D	Variable-amplitude displacement: The specimen was loaded in the tensile direction with displacement increments of 0.075 mm, up to a maximum displacement of 0.75 mm.
CL-DC-E	Variable-amplitude displacement: The specimen was loaded symmetrically with an amplitude increment of 0.075 mm, up to a maximum displacement of 0.6 mm.
CL-DC-G	Random displacement loading (simulating earthquake ground motion time history).
CL-DC-H	Random displacement loading (simulating seismic ground motion time history).
CL-SC-A	Constant amplitude strain: ±1%, ±2%, ±4%; each amplitude repeated 3 times.
CL-SC-D	Variable-amplitude strain: Unidirectional cyclic tensile loading with increments of 0.25% up to a maximum strain of 4.5%.

### 2.2. Salt Spray Test Setup

**Figure 2:**
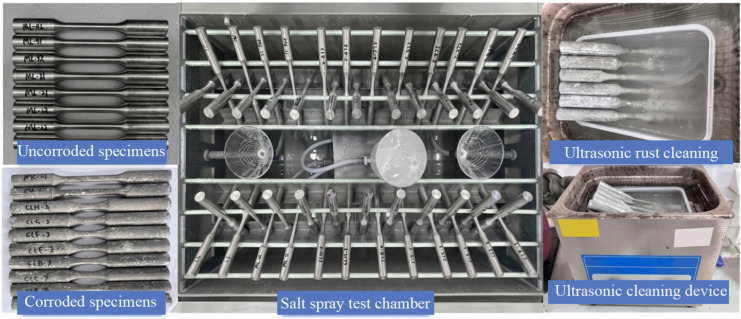
Accelerated salt spray corrosion test of aluminum alloy.

To simulate the high-temperature and high-salinity characteristics of coastal environments, this study conducted artificial accelerated corrosion tests on the specimens using a neutral salt spray test chamber, as shown in Figure 2, in accordance with the Chinese standard Corrosion tests in artificial atmospheres—Salt spray tests [22]. The experimental parameters are presented in Table 4.

To simulate the actual conditions of alternating wet and dry cycles in a coastal atmospheric environment, the salt spray test chamber was operated for 10 h daily to spray salt mist, while remaining inactive with the temperature reduced to room temperature for the rest of the day. All specimens were placed inside the chamber at an angle of 45° to the vertical and were flipped every 48 h to ensure uniform deposition of the corrosive medium on the specimen surfaces. Upon reaching the predetermined corrosion durations, the specimens were removed. Chemical and physical methods were employed to clear the corrosion products from the specimen surfaces, in accordance with the Chinese standard Corrosion of metals and alloys—Removal of corrosion products from corrosion test specimens [23]. Subsequently, the specimens were dried, weighed, and sealed for storage.

### 2.3. Loading Regimes

To systematically reveal the mechanical response and performance degradation mechanism of corroded aluminum alloys under different loading conditions, this study comprehensively adopted three methods: monotonic tensile displacement control, cyclic displacement control, and cyclic strain control. The objective was to obtain mechanical property parameters from two levels: the global response of the component and the constitutive relationship of the material.

The monotonic tensile tests were conducted on an MTS hydraulic servo testing machine using displacement control mode. To minimize experimental error, identical specimens (ML-1 and ML-2), as shown in Figure 1a, were used, and the average values of all key indicators were calculated. The loading regime was divided into two stages: a quasi-static rate of 0.1 mm/min was adopted when the stress was below the nominal yield strength of the material; once the stress reached the nominal yield strength, the loading rate was increased to 0.5 mm/min until specimen fracture.

**Figure 3:**
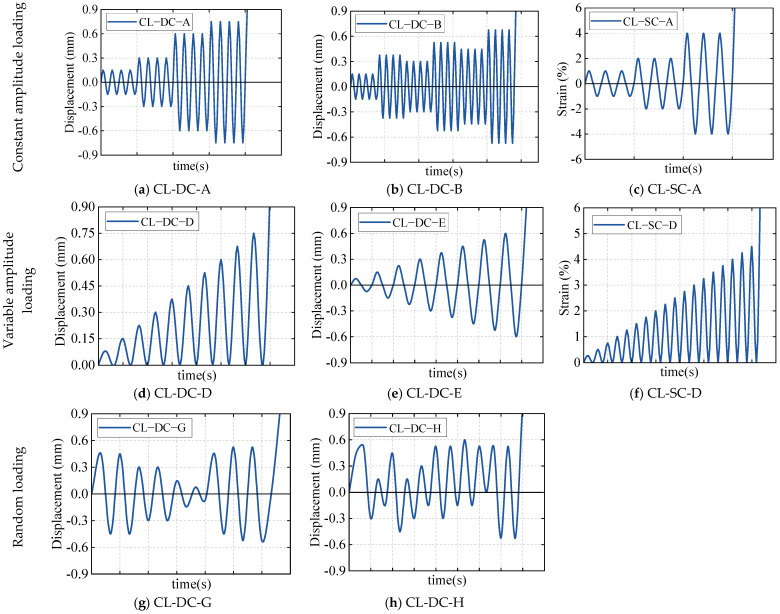
Cyclic loading regimes.

The cyclic loading tests aimed to reveal the hysteretic performance, energy dissipation capacity, and cumulative damage characteristics of corroded aluminum alloys under cyclic loads. Based on different research objectives, this study employed two modes, displacement control and strain control, with the cyclic loading regimes illustrated in Figure 3. The displacement-controlled loading mode was designed to simulate the global mechanical behavior of coastal structural components under common loads (such as wind vibration and ocean waves), directly evaluating the degradation of their stiffness, strength, and energy dissipation capacity. Combining the characteristics of actual service loads in coastal engineering, three displacement regimes were designed with reference to relevant national standards and measured data: constant-amplitude displacement (CL-DC-A, CL-DC-B), variable-amplitude displacement (CL-DC-D, CL-DC-E), and random displacement loading (CL-DC-G, CL-DC-H). The strain-controlled loading mode aimed to investigate the cyclic hardening behavior and stable stress–strain response of corroded aluminum alloys within a larger plastic strain range from the material constitutive level. Two strain loading regimes, constant-amplitude strain (CL-SC-A) and variable-amplitude strain (CL-SC-D), were designed with reference to the measured strain levels of aluminum alloy components under the action of strong earthquakes and extreme waves in coastal regions.

### 2.4. Test Implementation

**Figure 4:**
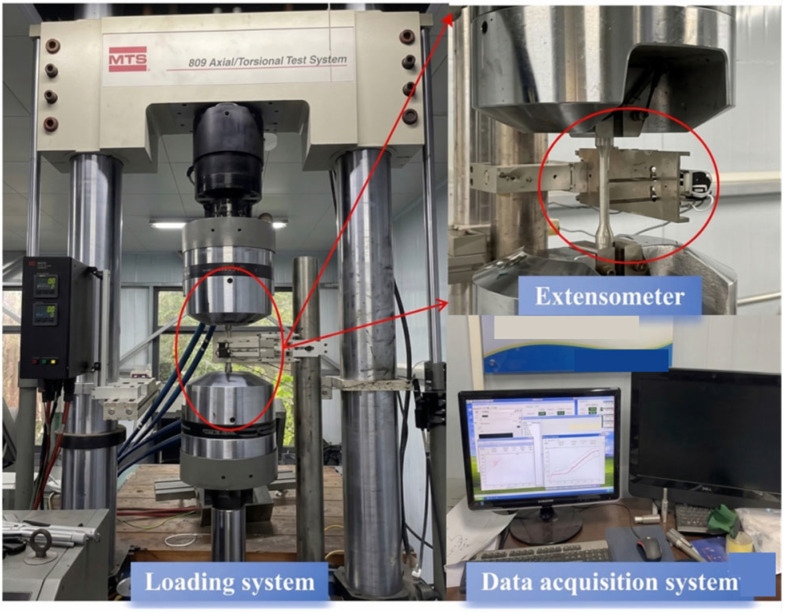
MTS servo-hydraulic test setup.

All cyclic loading tests were conducted on an MTS servo-hydraulic testing system, as illustrated in Figure 4. Displacement control was achieved via the built-in sensor of the actuator to establish closed-loop control, while strain control was implemented using an extensometer directly attached to the parallel length of the specimen for real-time monitoring and feedback. The loading process was executed in accordance with the Chinese standard Metallic materials—Tensile testing—Part 1: Method of test at room temperature [24]. The loading rate for displacement control was consistently set to 0.5 mm/min, and the strain rate for strain control was set to 0.001 s^−1^.

## 3. Results and Analysis

### 3.1. Tensile Properties

**Figure 5:**
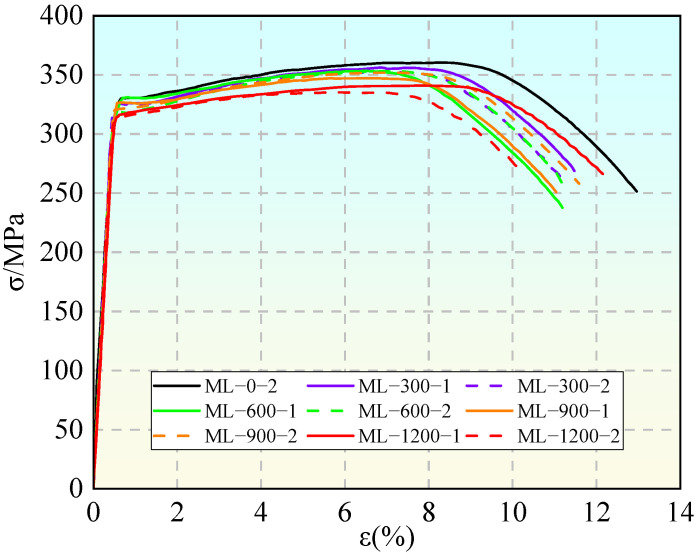
Tensile stress–strain curves under different corrosion durations.

**Figure 6:**
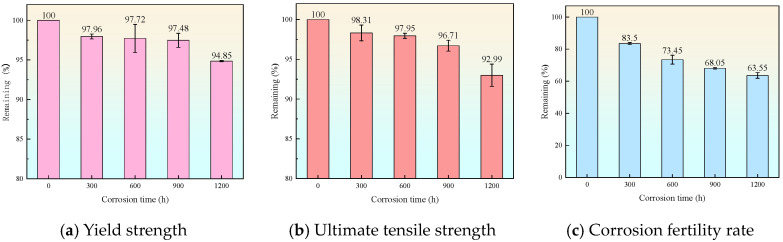
Degradation laws of mechanical properties of aluminum alloy under different corrosion durations.

Figure 5 and Figure 6 illustrate the degradation laws of the basic static properties of the aluminum alloy specimens under monotonic tension due to corrosion. As the corrosion degree increases, the engineering stress–strain curves of the material exhibit systematic deterioration: both yield strength and ultimate strength decrease with intensified corrosion. After 600 h of corrosion, the yield strength decreases by an average of approximately 2.28%; after 1200 h of corrosion, the ultimate strength decreases by an average of approximately 5.16%, and the peak stress point shifts noticeably to a lower strain level. Meanwhile, as the corrosion deepens, the ductility of the specimens shows a downward trend. The elongation after fracture significantly decreases, with a maximum reduction of about 13%, indicating that the plastic deformation capacity of the material is severely impaired. The mechanical property parameters of the corroded aluminum alloy are listed in Table 5.

The root cause of the aforementioned macroscopic performance degradation lies in the local stress concentration and deformation incompatibility induced by corrosion pits. Under tensile loading, the stress at the root of a pit is far higher than the average stress of the cross-section, causing this region to yield first and generate plastic strain, which results in a decrease in the macroscopic yield strength. Simultaneously, cracks preferentially initiate and propagate in the weakest regions with the most severe corrosion, leading to a rapid reduction in the effective load-bearing area. The fracture mode exhibits brittle characteristics, macroscopically manifesting as a shortened necking stage and reduced ultimate strain.

Although microscopic fractography was not conducted in this study, the macroscopic manifestation of a shortened necking stage and reduced ultimate strain strongly indicates a shift towards a pseudo-brittle fracture mode. Extensive literature on high-strength aluminum alloys [3,4] has confirmed that localized corrosion pits act as severe stress concentrators, leading to premature yielding and accelerated crack propagation.

### 3.2. Cyclic Loading

It should be noted that the cyclic loading results for the 300 h corrosion specimens showed negligible differences compared to the uncorroded (0 h) baseline. To maintain the conciseness of the manuscript and focus on the significant degradation stages, the 300 h cyclic data is omitted from the detailed discussion below.

#### 3.2.1. Hysteretic Performance and Damage Analysis Under Displacement-Controlled Loading

**Figure 7:**
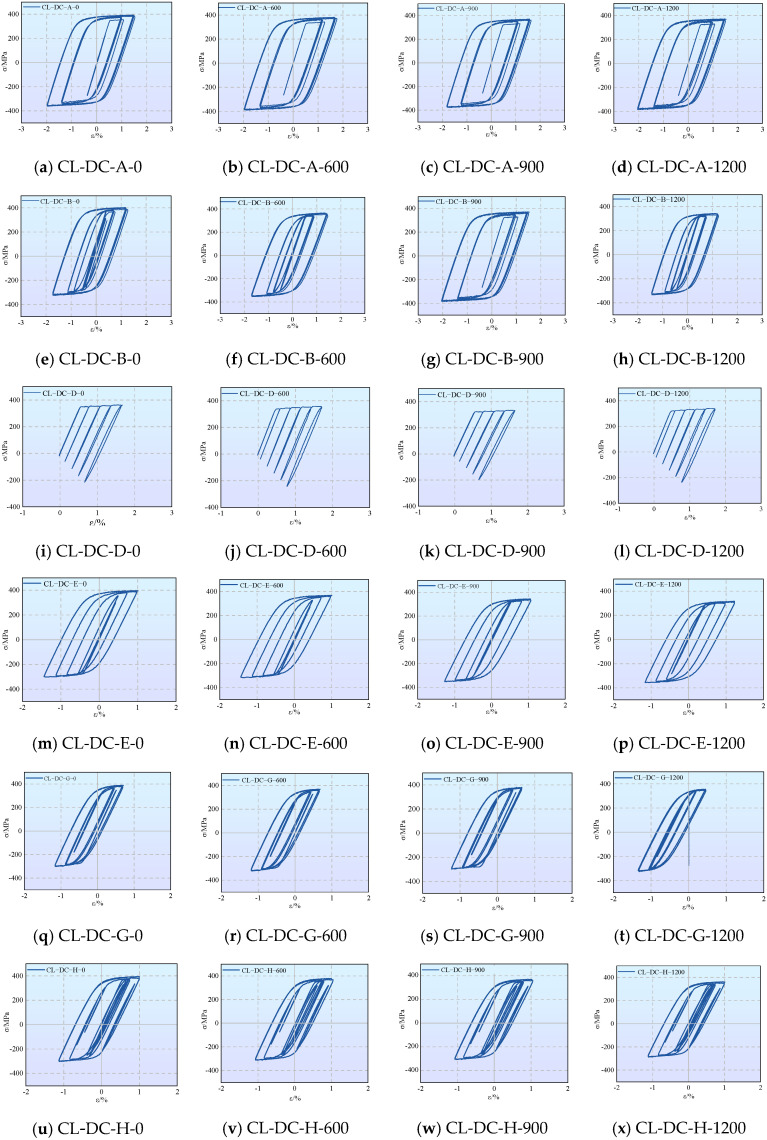
Hysteresis loops under cyclic loading (displacement control).

Figure 7 and Table 6 present the hysteresis loops (under displacement control) and mechanical parameters of specimens under different cyclic loading regimes. To quantitatively evaluate the degree of ductility degradation under combined corrosion and cyclic loading, a relative degradation parameter, denoted as ductility loss (*ƞ*), is formally introduced. It is calculated using Equation (1):

(1) $$\eta =\frac{E_{u,c}-E_{u,0}}{E_{u,0}}\times 100\%$$

where *E_u_*_,*c*_ represents the uniform elongation of the cyclically loaded specimen under specific corrosion conditions, and *E_u_*_,0_ is the uniform elongation of the uncorroded monotonic tensile baseline specimen (ML-0).

With increasing corrosion severity, the yield strength, ultimate strength, and elastic modulus of the material exhibit a systematic decline, and the cyclic hardening effect is significantly suppressed. Taking the CL-DC-A group as an example, the ultimate stress of the uncorroded specimen increased by 8.3% compared to the monotonic tensile baseline after cycling, whereas that of the specimen corroded for 1200 h remained essentially on par with the baseline.

Corrosion exacerbated the ductility degradation and the tendency toward brittle fracture. Compared with monotonic tension, all cyclically loaded specimens exhibited ductility deterioration, a process significantly accelerated by corrosion. For the CL-DC-A group, the ductility loss was 9.9% for the uncorroded specimen, which surged to 44.0% after 1200 h of corrosion.

Although direct microstructural characterization was not conducted in this study, the macroscopic manifestation of a shortened stable plastic deformation stage and a precipitous drop in ductility provides strong evidence of early crack initiation. Extensive literature on corroded high-strength aluminum alloys [9,11] has well established that localized corrosion pits act as severe stress concentrators and pre-existing crack sources. Under alternating cyclic stress, these pits facilitate rapid crack coalescence. This well-documented micro-mechanism perfectly explains the severe impairment of plastic deformation capacity and the pseudo-brittle fracture tendency observed in our tests.

The loading regime governed the damage accumulation mode. Under constant-amplitude (CL-DC-A, B) and regular-variable-amplitude (CL-DC-D, E) loading, damage accumulated in a stable and directional manner; ductility decreased in a regular pattern with intensifying corrosion, making the performance degradation highly predictable. Conversely, under random loading (CL-DC-G, H), due to the randomness of the load sequence, damage was triggered dispersedly across different microscopic regions. This resulted in more complex crack propagation and significant scatter in ductility degradation.

#### 3.2.2. Cyclic Response Characteristics Under Strain-Controlled Loading

**Figure 8:**
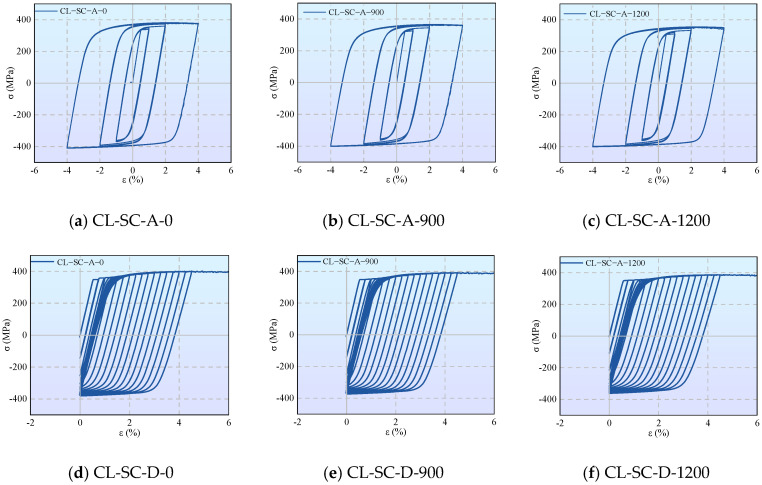
Hysteresis loops under cyclic loading (strain control).

The strain-controlled tests focus on the cyclic behavior of the material within a large plastic strain range. Figure 8 presents the hysteresis loops under cyclic loading (strain control). As observed from the figure, all specimens exhibit significant cyclic hardening; the stable stress amplitudes are far higher than the monotonic tensile yield strength. However, corrosion notably weakens the load-bearing capacity under large strains.

Symmetric constant amplitude loading (CL-SC-A) results in stable and symmetric hysteresis loops, with the skeleton curve displaying tension-compression symmetric saturation characteristics. In contrast, asymmetric variable-amplitude loading (CL-SC-D) exhibits a unidirectional Bauschinger effect. Corrosion primarily reduces the stress level corresponding to the same strain but does not significantly alter the plumpness of the hysteresis loops, indicating that the corroded aluminum alloy retains good energy dissipation capacity.

#### 3.2.3. Comparison of Monotonic and Cyclic Loading Test Results

**Figure 9:**
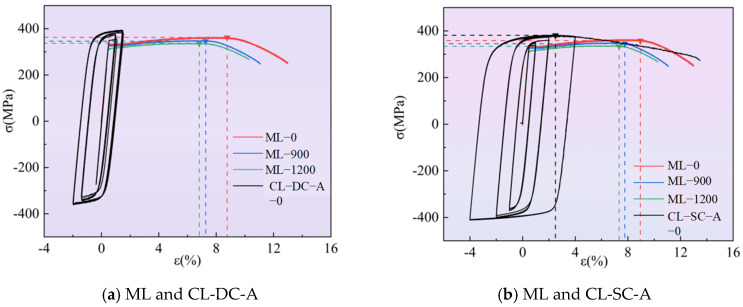
Engineering stress–strain curves under monotonic and cyclic loading.

The comparison of engineering stress–strain curves under monotonic and cyclic loading is illustrated in Figure 9. Influenced by the combined effect of cyclic hardening and the Bauschinger effect, the stress–strain curves of the aluminum alloy exhibit significant differences under monotonic and cyclic loading.

For uncorroded specimens, the ultimate strength after cycling increased by an average of 11% compared to the monotonic tensile baseline. However, this strengthening was achieved at the expense of deformability—the peak strain decreased by a maximum of 76%, causing the material to enter the necking stage earlier and significantly compressing the stable plastic deformation range.

With the deepening of corrosion, the strength increment induced by cyclic hardening gradually diminished or even vanished completely, while the degradation of peak strain became more pronounced. This indicates that the coupling of corrosion and cyclic loading is not a simple superposition of performance effects; rather, it accelerates the accumulation of internal damage within the material, driving the transition of its fracture mode from ductile to brittle.

#### 3.2.4. Skeleton Curves

**Figure 10:**
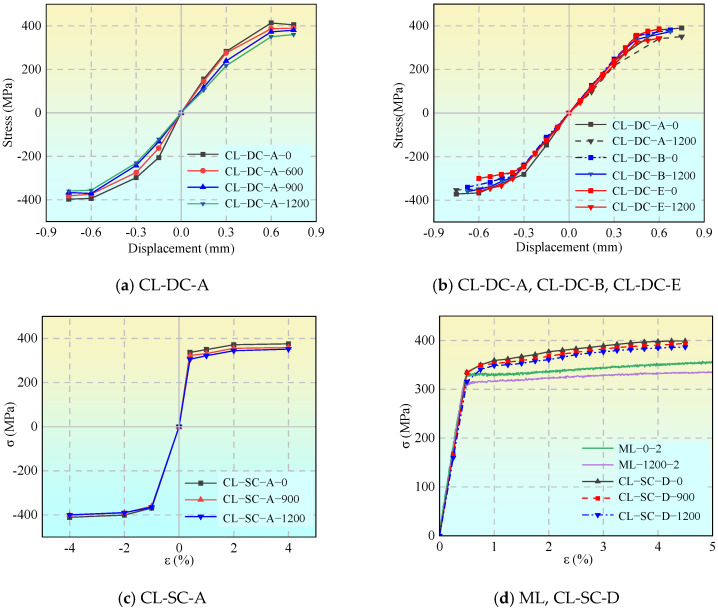
Skeleton curves under different loading regimes.

Figure 10 presents the skeleton curves under different loading regimes. At the same strain level, the stress values corresponding to cyclic loading are generally higher than those of monotonic loading, and the divergence between them becomes increasingly pronounced as the strain increases. This indicates that structural reorganization or dislocation strengthening has occurred within the material under reciprocating loads, exhibiting a significant cyclic hardening phenomenon. It implies that when subjected to repeated loads such as earthquakes, the cyclic response of the material is strongly dependent on the loading path, and unidirectional tension-dominated loading will gradually mobilize its hardening capacity until saturation.

#### 3.2.5. Energy Dissipation

**Figure 11:**
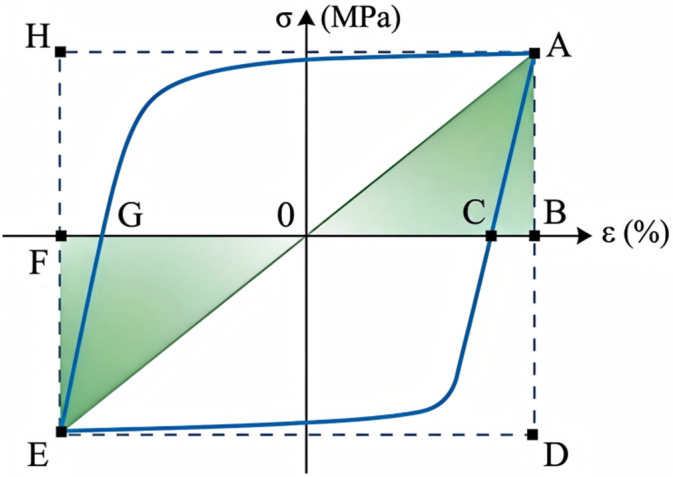
Calculation method of energy dissipation coefficient.

The energy dissipation coefficient [25] is employed to evaluate the energy dissipation capacity of the aluminum alloy, and its calculation method is illustrated in Figure 11. A larger energy dissipation coefficient corresponds to a plumper hysteresis loop, indicating a stronger energy dissipation capacity of the metal.

(2) $$E_{e}=\frac{S_{AGC}+S_{ECG}}{S_{\Delta AOB}+S_{\Delta EOF}}$$

where *S_AGC_* and *S_ECG_* represent the areas of the hysteresis curve above and below the horizontal axis, respectively. *S*_Δ*AOB*_ and *S*_Δ*EOF*_ are the areas of the triangles located above and below the horizontal axis. A higher energy dissipation coefficient indicates a fuller hysteresis loop, signifying a stronger energy dissipation capacity of the material.

Based on the data of cumulative energy dissipation (Table 7) and energy dissipation coefficient (Table 8), the effects of corrosion and loading history on the energy dissipation performance of the aluminum alloy are evaluated from three aspects: total energy dissipation, efficiency, and evolution process.

Under all loading regimes, both the cumulative energy dissipation and energy dissipation coefficient of corroded specimens are lower than those of uncorroded ones. For DC-A specimens under displacement-controlled constant-amplitude loading, the total cumulative energy dissipation of the specimens corroded for 1200 h decreased by approximately 9% compared to the uncorroded specimens. For SC-A specimens under strain-controlled loading, although the percentage reduction was only 3%, the absolute loss reached 712 J, indicating a prominent loss of energy dissipation potential during the large deformation stage.

Under constant amplitude loading, the evolution of energy dissipation performance is gradual and highly predictable. In contrast, variable amplitude and random loading, due to their complex loading histories, accelerated the damage process, leading to earlier specimen failure and severe fluctuations in energy dissipation parameters. This underscores the degradation risk posed by actual random loads to corroded structures.

The cumulative energy dissipation and energy dissipation coefficient under strain control are significantly higher than those under displacement control. When the cyclic loading amplitude of SC-A specimens reaches 4%, the energy dissipation coefficient stabilizes between 3.0 and 3.3. Only when the 6061-T6 aluminum alloy fully enters the plastic stage can it exert excellent energy dissipation capacity.

**Figure 12:**
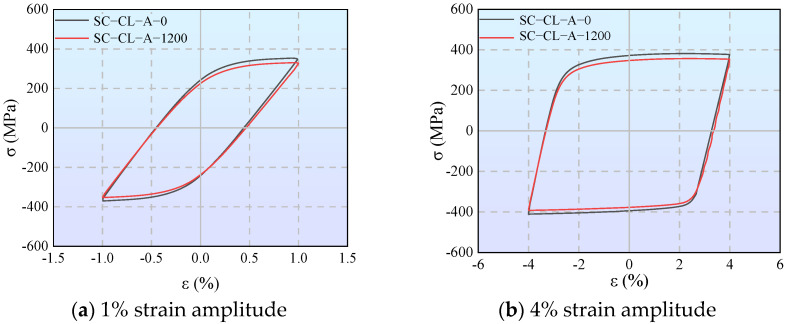
Comparison of hysteresis loop morphologies at different strain amplitudes.

Figure 12 compares the morphologies of hysteresis loops for specimen SC-CL-A with different corrosion periods under strain amplitudes of 1% and 4%. It is evident that the hysteresis loops of the uncorroded specimen remained consistently plump, whereas the loop area of the corroded specimen gradually decreased as cycling progressed. For instance, at a strain amplitude of 4%, the energy dissipated per cycle decreased by approximately 5%, and the energy dissipation coefficient dropped by about 7%. This indicates that corrosion not only degrades the load-bearing capacity and stiffness of the component but also severely impairs its capacity to absorb energy through plastic deformation, posing a direct threat to the seismic safety of the structure.

Based on the obtained degradation laws, practical engineering designs for coastal aluminum structures should explicitly account for the time-dependent synergistic effects of corrosion and cyclic loads. Firstly, for strength-governed designs, time-variant reduction factors must be incorporated into the calculation of load-bearing capacity. Secondly, regarding ductility, the severe ductility loss under cyclic loads indicates that traditional ductility-based seismic design provisions may dangerously overestimate the deformation capacity of corroded components. Therefore, designers should strictly limit the allowable plastic strain amplitude and enhance structural redundancy to prevent sudden pseudo-brittle failures. Finally, considering the decay in energy dissipation, the structural damping ratio will inevitably decrease over time. For critical joints exposed to random dynamic loads, supplementary energy dissipation devices or appropriately increased sacrificial cross-sectional thicknesses should be considered to guarantee long-term service safety.

## 4. Conclusions

In this study, the 6061-T6 aluminum alloy, commonly used in coastal engineering, was investigated. Through laboratory accelerated salt spray corrosion tests, monotonic tensile tests, and multi-regime cyclic loading tests, the effects of varying corrosion degrees on the material’s static properties, cyclic hysteretic characteristics, and energy dissipation capacity were explored. The main conclusions are as follows:

(1) After 600 h of corrosion, the yield strength decreased by an average of approximately 2.28%; after 1200 h of corrosion, the ultimate strength decreased by an average of approximately 5.16%, and the peak stress point shifted noticeably to a lower strain level. Simultaneously, as corrosion severity increased, the ductility of the specimens exhibited a declining trend. The elongation after fracture decreased significantly, with a maximum reduction of about 13%, indicating that the plastic deformation capacity of the material was severely impaired.
(2) The coupling effect of corrosion and cyclic loading dominates the degradation of cyclic performance. Corrosion significantly suppresses the cyclic hardening effect; for specimens corroded for 1200 h, the cyclic strengthening effect essentially vanished. Meanwhile, corrosion accelerates ductility loss; the severely corroded specimens in the CL-DC-A group experienced a ductility loss of up to 44.0%. Under constant amplitude and regular variable amplitude loading, performance degradation shows high predictability, whereas random loading results in significant scatter.
(3) Strain-controlled tests confirmed that the weakening of mechanical properties by corrosion persists throughout the entire process from elastic to fully plastic deformation. Within the large strain range, the material exhibits significant cyclic hardening, yet corrosion reduces its load-bearing capacity. At a strain amplitude of ±4%, the stable stress amplitude decreased from 376 MPa (uncorroded) to 350 MPa. However, corrosion did not significantly alter the plumpness of the hysteresis loops, suggesting the material still retains good energy dissipation potential.
(4) The energy dissipation capacity of the 6061-T6 aluminum alloy is jointly governed by corrosion, loading regime, and strain level. Corrosion led to a significant decline in cumulative energy dissipation; for DC-A specimens under displacement-controlled constant amplitude loading, the total cumulative energy dissipation of specimens corroded for 1200 h decreased by 9%. When the cyclic xqstrain amplitude of SC-A specimens reached 4%, the energy dissipation coefficient stabilized between 3.0 and 3.3. Variable amplitude and random loading accelerated the damage process and exacerbated the risk of performance degradation.

## Figures and Tables

**Table 4:** Experimental conditions of the neutral salt spray chamber.

Experiment Item	Experimental Condition
Experimental temperature	35 °C
Experiment humidity	93 ± 3
NaCl solution	5% (wt.%)
pH	6.5~7.5
Spray mode	Continuous spray
Salt spray deposition rate	1~2 mL/(80 cm^2^·h)

**Table 5:** Mechanical property parameters of corroded aluminum alloy.

Specimen Labels	*ρ*/(%)	*E*/GPa	*ε*/(%)	*f*_y_/MPa	Δ*f*_y_	*f*_u_/MPa	Δ*f*_u_
ML-0-1	-	70.4	12.49	331.2	0.00%	362.4	0.00%
ML-0-2	-	71.5	12.96	330.2		363.6	
ML-300-1	0.169	70.5	11.49	323.7	−2.04%	358.8	−1.69%
ML-300-2	0.161	71.2	11.34	324.2		354.9	
ML-600-1	0.246	66.7	11.19	318.5	−2.28%	355.8	−2.05%
ML-600-2	0.285	69.9	11.20	327.8		355.3	
ML-900-1	0.323	68.4	11.04	324.9	−2.52%	348.7	−3.29%
ML-900-2	0.316	68.1	11.67	319.8		353.4	
ML-1200-1	0.352	67.6	12.26	314.3	−5.15%	340.6	−7.01%
ML-1200-2	0.377	66.3	10.32	313.0		334.5	

Note: *ρ* is the corrosion rate; *E* is the modulus of elasticity; *ε* is the elongation; *f*_y_ is the yield strength (since aluminum alloys do not exhibit a distinct yield point, the stress corresponding to a 0.2% non-proportional extension is typically defined as the nominal yield strength in most engineering applications and material standards.); Δ*f*_y_ is the change in yield strength; *f*_u_ is the ultimate tensile strength; Δ*f*_u_ is the change in ultimate strength.

**Table 6:** Results of loading tests.

Specimens	*σ*_y_/MPa	*σ*_u_/MPa	*V*/%	*σ*_u_/*σ*_y_	*Ε*_u_*/*%	*η*/%
ML-0	331	363	0	1.10	12.9	0
CL-DC-A-0	345	393	8.3	1.14	11.6	−9.9
CL-DC-A-600	329	381	5.0	1.16	11.1	−14.3
CL-DC-A-900	320	372	2.5	1.16	9.5	−26.5
CL-DC-A-1200	315	362	−0.3	1.15	7.2	−44.0
CL-DC-B-0	331	398	9.6	1.20	9.5	−26.0
CL-DC-B-600	326	379	4.4	1.16	12.3	−4.5
CL-DC-B-900	322	366	0.8	1.14	12.8	−0.6
CL-DC-B-1200	317	356	−1.9	1.12	9.7	−24.9
CL-DC-D-0	340	372	2.5	1.09	13.6	5.6
CL-DC-D-600	331	368	1.4	1.11	13.7	6.1
CL-DC-D-900	323	342	−5.8	1.06	14.2	9.9
CL-DC-D-1200	321	344	−5.2	1.07	13.5	5.0
CL-DC-E-0	331	404	11.3	1.22	11.4	−11.6
CL-DC-E-600	326	377	3.9	1.16	12.4	−3.9
CL-DC-E-900	320	360	−0.8	1.13	9.8	−23.8
CL-DC-E-1200	311	340	−6.3	1.09	8.9	−30.9
CL-DC-G-0	331	395	8.8	1.19	12.4	−4.1
CL-DC-G-600	323	381	5.0	1.18	13.3	2.8
CL-DC-G-900	323	385	6.1	1.19	11.9	−7.6
CL-DC-G-1200	307	365	0.6	1.19	13.4	3.7
CL-DC-H-0	337	395	8.8	1.17	12.5	−3.2
CL-DC-H-600	327	380	4.7	1.16	13.2	2.1
CL-DC-H-900	321	371	2.2	1.16	12.1	−6.5
CL-DC-H-1200	315	366	0.8	1.16	12.9	−0.3

Note: *σ*_y_ is the yield strength; *σ*_u_ is the ultimate tensile strength; *V* is the percentage increase in yield strength; *σ*_u_/*σ*_y_ is the tensile-to-yield ratio; *Ε*_u_ is the uniform elongation; *ƞ* is the ductility loss.

**Table 7:** Cumulative energy dissipation (Unit: J).

Energy Dissipation	DC-A −0	DC-A −1200	DC-E −0	DC-E −1200	DC-G −0	DC-G −1200	SC-A −0	SC-A −1200
cycle1	227	200	186	164	165	136	584	511
cycle2	232	203	197	188	169	144	588	514
cycle3	237	206	216	195	189	174	591	518
cycle4	423	395	-	-	193	178	1982	1904
cycle5	422	392	-	-	-	-	2006	1917
cycle6	422	391	-	-	-	-	2015	1929
sum	1964	1787	599	547	716	632	22,741	22,029

**Table 8:** Energy dissipation coefficient.

Energy Dissipation Coefficient	DC-A −0	DC-A −1200	DC-E −0	DC-E −1200	DC-G −0	DC-G −1200	SC-A −0	SC-A −1200
cycle1	1.11	0.95	0.25	0.21	0.28	0.21	1.73	1.55
cycle2	1.07	0.94	0.57	0.54	0.24	0.18	1.71	1.66
cycle3	1.06	0.92	0.88	0.86	0.65	0.48	1.66	1.65
cycle4	1.52	1.41	-	-	0.71	0.53	2.64	2.22
cycle5	1.52	1.39	-	-	-	-	2.63	2.24
cycle6	1.49	1.35	-	-	-	-	2.6	2.31
cycle-7	-	-	-	-	-	-	3.23	3.05
cycle-8	-	-	-	-	-	-	3.21	3.07
cycle-9	-	-	-	-	-	-	3.24	3.11

## Data Availability

The original contributions presented in this study are included in the article. Further inquiries can be directed to the corresponding author.
